# Supplementary material for: Clinically relevant pathogens on surfaces display differences in survival and transcriptomic response in relation to probiotic and traditional cleaning strategies
Source: NPJ Biofilms Microbiomes. 2022 Sep 19;8:72. doi: 10.1038/s41522-022-00335-7 (PMC9485146; doi:10.1038/s41522-022-00335-7)
Supplement: Supplementary file 1 — Supplementary Information [file 41522_2022_335_MOESM1_ESM.pdf]

Supporting Information

Clinically Relevant Pathogens on Surfaces Display Differences in Survival and Transcriptomic Response in Relation to Probiotic and Traditional Cleaning Strategies

Jinglin Hu, Weitao Shuai, Jack T. Sumner, Anahid A. Moghadam, Erica M. Hartmann*

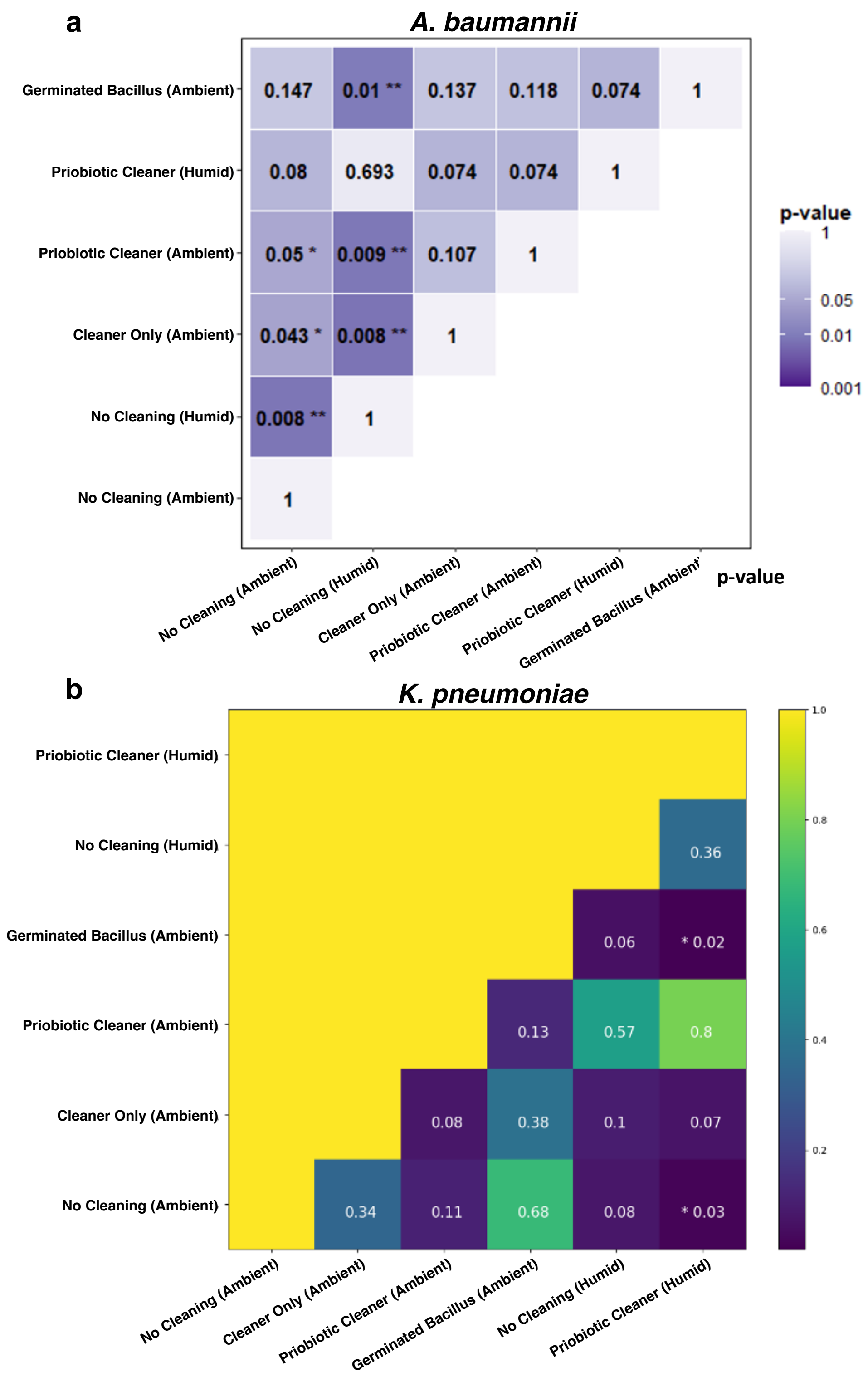
a
A. baumannii
Germinated Bacillus (Ambient)
Priobiotic Cleaner (Humid)
Priobiotic Cleaner (Ambient)
Cleaner Only (Ambient)
No Cleaning (Humid)
No Cleaning (Ambient)
0.147 0.01 ** 0.137 0.118 0.074 1
0.08 0.693 0.074 0.074 1
0.05 * 0.009 ** 0.107 1
0.043 * 0.008 ** 1
0.008 ** 1
1
No Cleaning (Ambient)
No Cleaning (Humid)
Cleaner Only (Ambient)
Priobiotic Cleaner (Ambient)
Priobiotic Cleaner (Humid)
Germinated Bacillus (Ambient
p-value
p-value
1
0.05
0.01
0.001
b
K. pneumoniae
Priobiotic Cleaner (Humid)
No Cleaning (Humid)
Germinated Bacillus (Ambient)
Priobiotic Cleaner (Ambient)
Cleaner Only (Ambient)
No Cleaning (Ambient)
0.36
0.06 * 0.02
0.13 0.57 0.8
0.08 0.38 0.1 0.07
0.34 0.11 0.68 0.08 * 0.03
No Cleaning (Ambient)
Cleaner Only (Ambient)
Priobiotic Cleaner (Ambient)
Germinated Bacillus (Ambient)
No Cleaning (Humid)
Priobiotic Cleaner (Humid)
1.0
0.8
0.6
0.4
0.2

Supplementary Figure 1. p-values of *A. baumannii* (A) and *K. pneumoniae* (B) viable CFU counts among samples collected under two temperature/humidity conditions and four cleaning scenarios. Statistical testing was conducted using Welch's paired t-test. * $p<0.05$, ** $p< 0.01$, and ***$p < 0.001$.

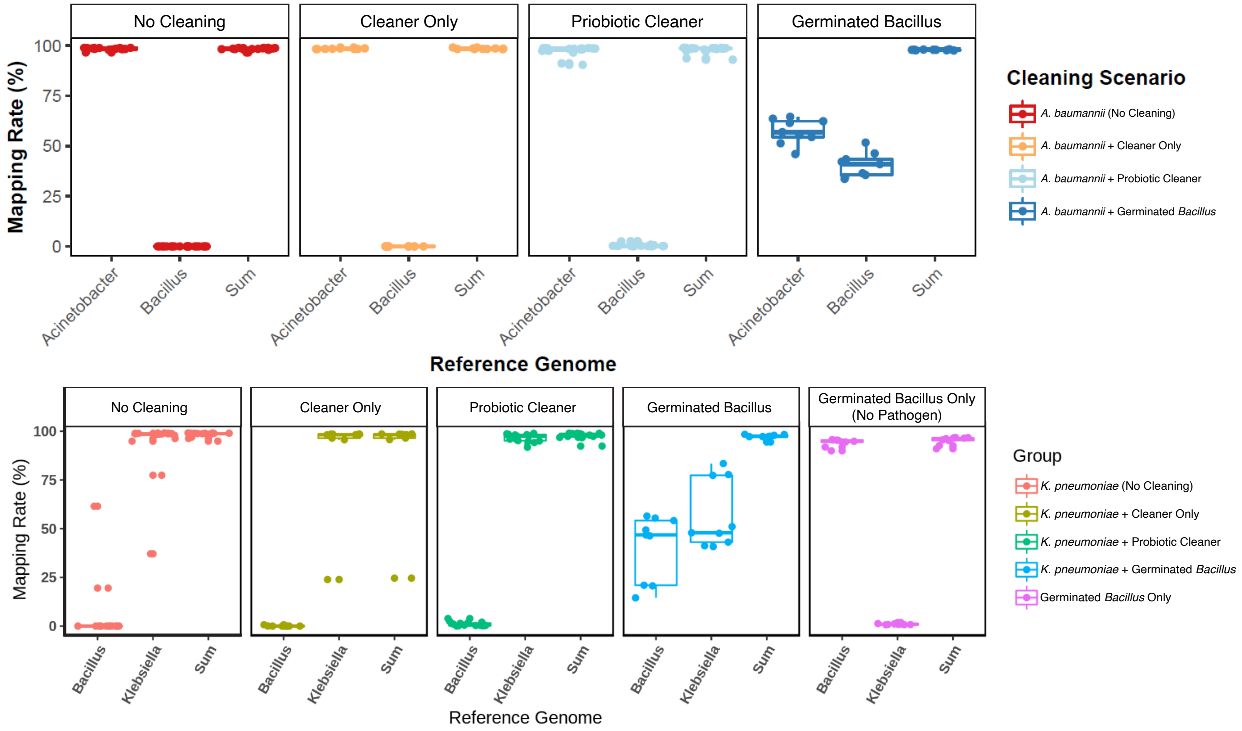

Supplementary Figure 2. Percentages of reads mapped onto *Bacillus* spp. and either *A. baumannii* (top) or *K. pneumoniae* (bottom) genomes for samples collected from four surface cleaning scenarios.

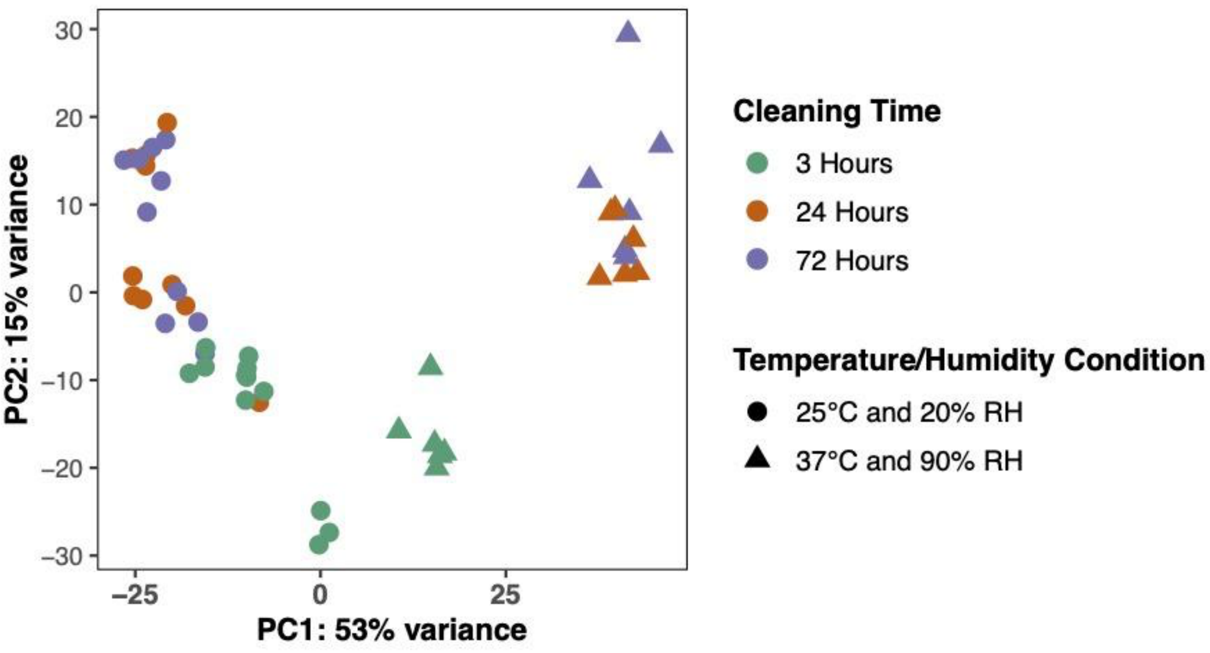

Supplementary Figure 3. Principal components analysis on variance stabilized *K. pneumoniae* read counts. Samples were color-coded based on cleaning time.

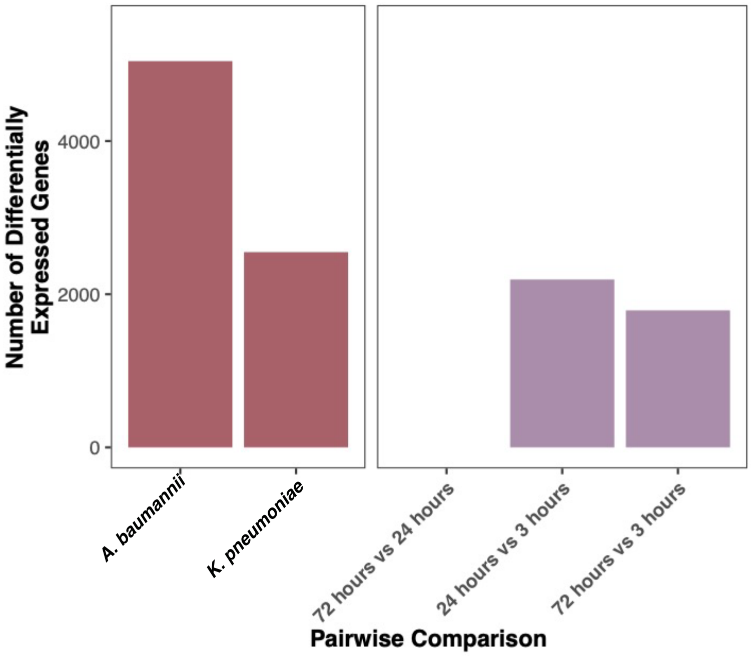

Supplementary Figure 4. Number of differentially expressed genes for germinated *Bacillus* reads. The *Bacillus* transcriptome was compared in the presence of *A. baumannii* or *K. pneumoniae* using vegetative *Bacillus* without pathogen as the reference level. Comparison was also conducted between different time points.

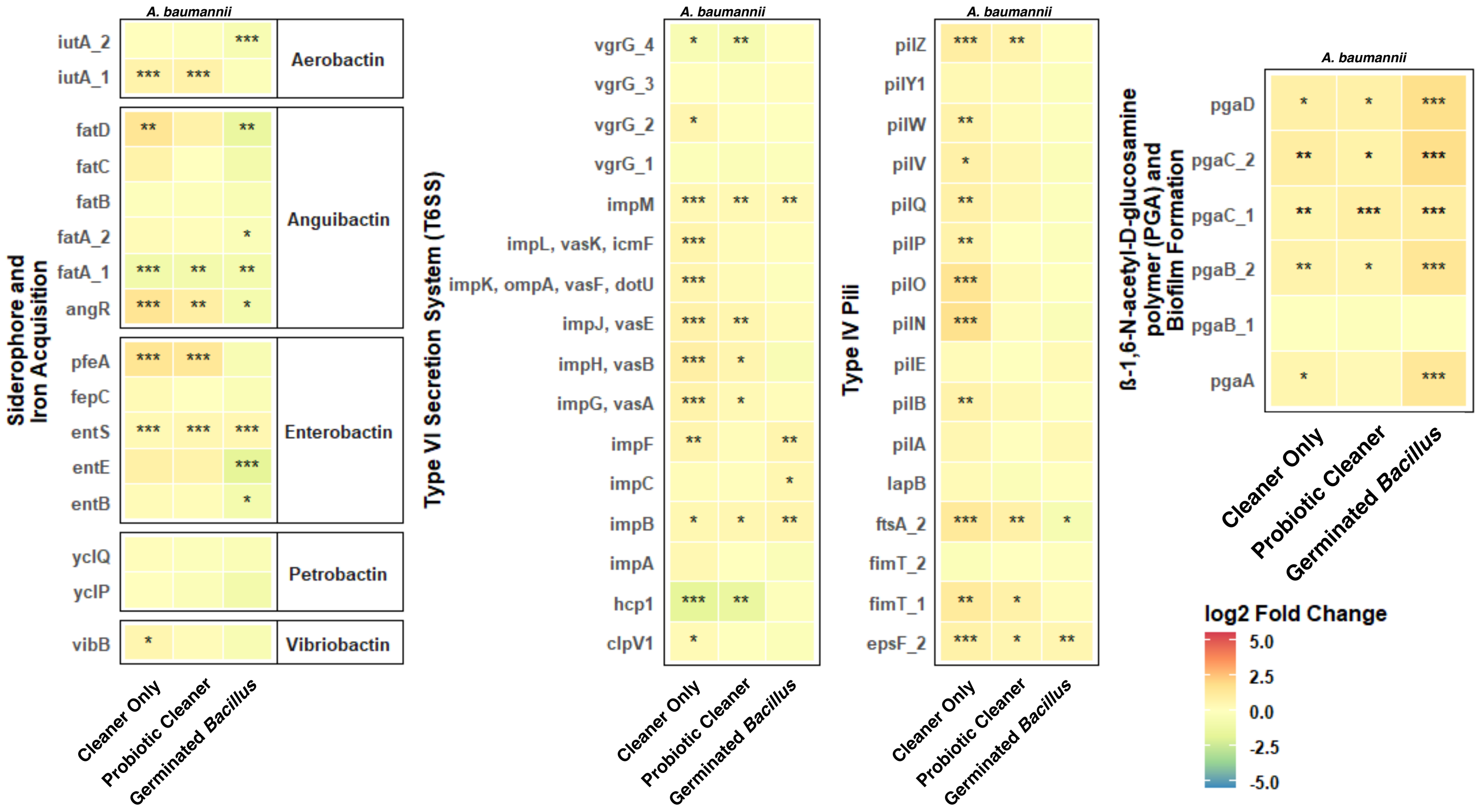

Supplementary Figure 5. Log_2 fold change of genes associated with iron acquisition, Type VI Secretion System (T6SS), Type VI Pili, and biofilm poly-beta-1,6-N-acetyl-D-glucosamine (PGA) production in samples cleaned with Cleaner Only, Probiotic Cleaner, and Germinated *Bacillus* compared to No Cleaner samples (*A. baumannii* only). Statistically significant log_2 fold changes were determined by DESeq2 with Benjamini-Hochberg corrected p-value ≤ 0.05. * $p<0.05$, ** $p< 0.01$, and ***$p < 0.001$.

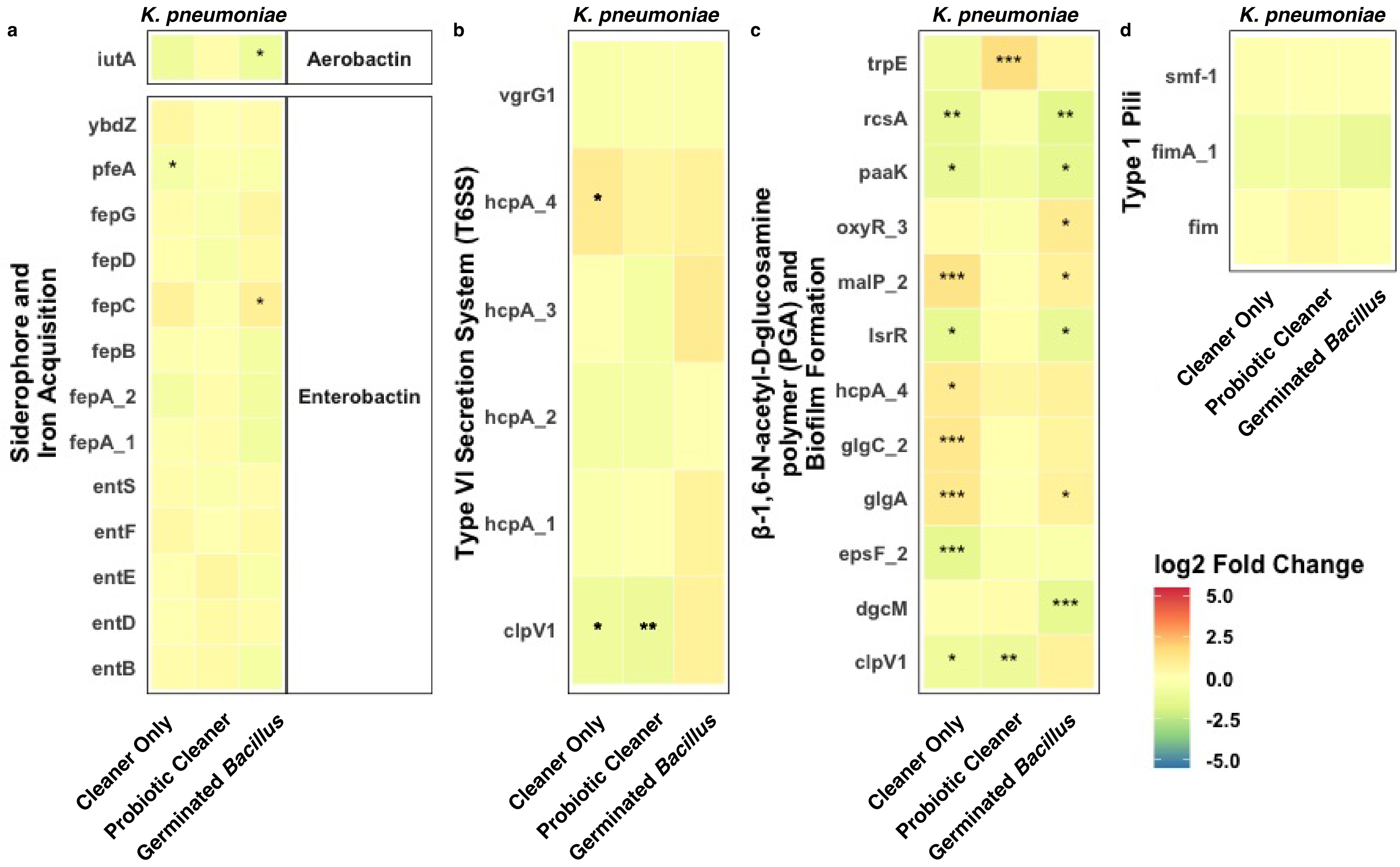

Supplementary Figure 6. Log$_2$ fold change of genes associated with iron acquisition, Type VI Secretion System (T6SS), biofilm poly-beta-1,6-N-acetyl-D-glucosamine (PGA) production, and Type 1 Pili in samples cleaned with Cleaner Only,

Probiotic Cleaner, and Germinated *Bacillus* compared to No Cleaner samples (*K. pneumoniae* only). Statistically significant log_2 fold changes were determined by DESeq2 with Benjamini-Hochberg corrected p-value ≤ 0.05. Some genes have Benjamini-Hochberg corrected p-value ≤ 0.05 but log_2 fold change < 1. * $p<0.05$, ** $p< 0.01$, and ***$p < 0.001$.

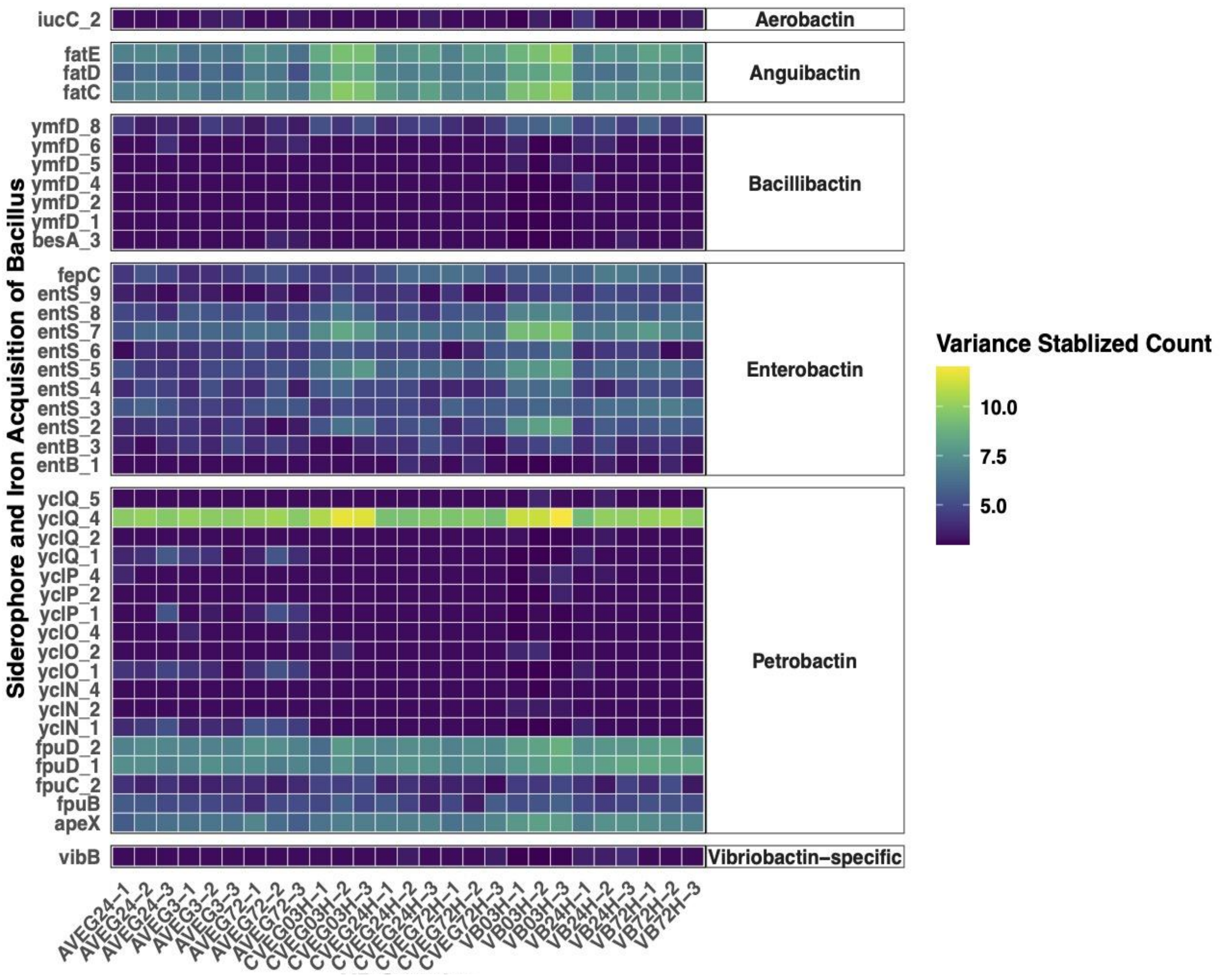

Supplementary Figure 7. Expression of genes associated with siderophore and iron acquisition in vegetative *Bacillus* spp. samples in the presence of *A. baumannii* (AVEG), *K. pneumoniae* (CVEG), and in the absence of pathogen (VB).

Read counts belonging to Bacillus *spp*. were transformed using variance stabilization to enable between-sample comparisons.
